# African Swine Fever and Its Control Measures in Wild Boar: A *“De Iure Condito”* Analysis in the European Union

**DOI:** 10.3390/ani14010014

**Published:** 2023-12-19

**Authors:** Sergio Migliore, Hany A. Hussein, Paola Galluzzo, Roberto Puleio, Guido Ruggero Loria

**Affiliations:** Istituto Zooprofilattico Sperimentale della Sicilia “A. Mirri”, 90129 Palermo, Italy; sergio.migliore@izssicilia.it (S.M.); hany.ahmed@izssicilia.it (H.A.H.); paola.galluzzo@izssicilia.it (P.G.); guidoruggero.loria@izssicilia.it (G.R.L.)

**Keywords:** African swine fever, wild boar, porcine animals, animal health law, European Union law

## Abstract

**Simple Summary:**

The arrival of the African swine fever (ASF) epizootic in the European continent has pushed public veterinary authorities to act immediately in order to contain its spread by applying the regulatory guidance of the current Animal Health Law of the European Union (Regulations EU 2016/429). Control and surveillance measures, in accordance with Regulations 2020/687 and 2023/594, have been implemented. Moreover, protection and surveillance areas have been created around the infected holdings and the wild boar cases. This paper analyzes the advantages and critical points of the legal framework for the control of ASF in wild boar, focusing on the role of official veterinary services in European Union Member States (EU MS).

**Abstract:**

Recently, the African swine fever (ASF) epizootic has been reported in domestic pigs and wild boars in several European Union Member States (EU MS) and epidemiological evidence has accumulated which indicates that wild boar play a key role in maintaining and spreading the disease. Thanks to the experience gained when managing ASF outbreaks in Sardinia (Italy) and Eastern Europe, Directive 2002/60 CE was issued. This directive represented an important step forward in controlling the disease, particularly the risk of spreading the virus to wild animals. Since 2021, according to Regulation (EU) 2016/429, which is also called “Animal Health Law—AHL”, when the MS competent authority suspects or confirms ASF (a cat. A listed disease) in wild animals, it is mandatory to conduct surveillance in the wild boar population and implement the necessary control measures. Within AHL, Regulations (EU) 2020/687 and 2023/594 established special ASF control measures in kept and wild porcine animals, and their products and by-products, focusing on and underlying old and new responsibilities that vets (both public and private ones) have to accomplish under the new regulations. The new change in the legal framework deals with specific measures to be applied in the wild and represents a great challenge for MS veterinary services. Some of these measures have been well established in the last two decades, particularly those related to application in the farming system, while other measures are still new to veterinary health management and require a holistic approach in terms of intensity, considering all geographical, ecological, productive, cultural and social features of the involved EU MS. In this contribution, the authors intend to focus on specific measures which have been issued in order to limit or stop the spread of ASF in a wild, “boundless” ecosystem. These measures expand the field of competence of the official veterinary service to wild areas in addition to farm activity.

## 1. Introduction

The European Union (EU) Regulation 2016/429, commonly known as the “Animal Health Law (AHL)”, is a single reference law covering all aspects of animal health in the European Union Member States (EU MS) [1]. The AHL applies to domestic (kept) and wild animals, and categorizes “notifiable” (now “listed”) diseases according to their level of epidemiological risk and specified control measures. This simplifies practical procedures, tasks and responsibilities to prevent or limit the spread of animal diseases [2].

According to the AHL, when suspecting or confirming that a listed disease has occurred in wild animals, the EU MS competent authority must conduct surveillance in the target wild animal population, implementing the necessary disease control measures [1].

African swine fever (ASF) is a highly contagious and lethal viral disease caused by African swine fever virus (ASFV) that affects domestic and wild pigs of all ages [3], with serious economic impacts on the swine industry. ASF belongs to the highest-impact listed diseases (category A).

Out of 23 different ASFV genotypes circulating in Africa [4], only genotypes I and II have been described in Europe. Genotype I was first detected in Europe in 1957 [5], with successive epizootics until the 1990s [6], while genotype II was detected for the first time in 2007 in the Russian Federation [7] and re-emerged in the eastern EU MS in 2014, where it became endemic in wild boar [8]. Outbreaks were then reported in Belgium in 2018 [9], in Germany in 2020 [10] and more recently in Italy [11] and Greece [12]. The epidemiology of ASF is very complex and four epidemiological cycles have been identified [13]. This epidemiologic complexity can result in different scenarios depending on geographical areas, species involved, routes of transmission and risk factors, linked to persistence and spread of the virus in the environment [4]. Inter-cycle disease transmission may occur and result in spread of the infection to the wild boar habitat, which seems to be the causative factor for long-distance spread of ASF. Therefore, it is essential to sustain and enlarge the geographical range of the transmission cycle in wild boar habitat for proper control of the ongoing epizootic [13].

Today, wild boar have been recognized as the main potential long-term hosts of ASFV, independently from pigs and ticks in all EU MS, and the virus circulation in wild ecosystems is the main risk of infection spreading throughout EU MS [14,15,16]. Wild boar ecology and behavior may facilitate the spread of ASFV due to their high mobility, sociality and reproductive potential [16]. Positive associations between wild boar population density and ASF have been found [14], though not predicted previously [17].

The spread of ASF in wild boar in EU MS mostly occurs in an “oil spot” modality, due to their extensive movements (up to 40 km in a straight line) in boundless ecosystems (not having the physical limitations of a livestock farm) and the possibility of moving to a wide range of areas and habitats, therefore spreading ASFV in unpredictable areas [18]. High hunting pressure and other outdoor human activities are deemed to substantially intensify wild boar dispersal movements, which subsequently helps the spread of ASFV in the EU MS [16].

These epidemiologic drivers of disease intermingle with wild boar population determinants such as wild boar demography, management factors, hunting rates and hunting techniques [13].

At the moment, the main challenge of ASF control in the EU MS is precisely represented by the control of unpredictable wild boar movements, representing the main risk factor in the spread of the disease. The arrival of the ASF epizootic in EU MS has forced public veterinary authorities to act immediately in order to counteract its spread, applying the regulatory guidance of the AHL.

This paper aims to comment on the advantages and critical points of the AHL and related EU Commission Implementing Regulations (CIR) for the control of ASF in wild boar in EU MS, focusing on the roles of the official veterinary services.

## 2. The New Rules Applied to the Complex Epidemiology of ASF

According to article no. 5 of the AHL, ASF belongs to category A diseases because of its epidemiological characteristics [1]. CIR (EU) 2023/594 [19] repeals Implementing Regulation (EU) 2021/605 [20], laying down special ASF control measures in farmed pigs and wild boar, their products and by-products adopted within the framework of Regulation (EU) 2016/429, specifically the following:

○ ANNEX I defines the restricted zones I, II and III at union level;
○ ANNEX II defines the areas at union level as infected or restricted zones, including protection and surveillance zones. In particular, following an outbreak of ASF in wild boar, areas are established as infected zones. In contrast, in farmed pigs, outbreaks areas are established as restricted zones, comprising protection and surveillance zones;
○ ANNEX IV defines minimum requirements for national action plans for wild boar in order to avoid the spreading of ASF in EU MS, based on the level of epidemiological risk and keeping into account the logistic, ecological, social and productive situation surrounding the suspected or confirmed case. In particular, national action plans must:
  ○ Establish the objectives, priorities and goals of the national action plan, with a description of scientific measures set out in the plan;
  ○ Describe the roles and functions of the main institutions and stakeholders;
  ○ Estimate, with valid scientific methods, the population size of wild boar;
  ○ Describe hunting management;
  ○ Describe targets and measures used to control and reduce wild boar populations;
  ○ Describe biosecurity requirements related to hunting of wild boar;
  ○ Describe union or national biosecurity measures to protect farmed pigs from wild boar;
  ○ Implement arrangements, including a timetable for different measures;
  ○ Provide a communication strategy for hunters and training campaigns concerning ASF risk to prevent the introduction and dissemination of the disease by hunters;
  ○ Provide a joint program of cooperation between agricultural and environmental boards;
  ○ Describe cross-border cooperation with other MS and third countries;
  ○ Describe the compulsory, continuous, passive surveillance testing of wild boar;
  ○ Assess the possible negative effects of hunting activities.

Within 90 days from the first outbreak, the competent authority has to prepare an eradication plan to be immediately transmitted to the EU Commission for approval before its application.

Annexes I and II of CIR (EU) 2023/594 are constantly amended and updated according to the evolution of the European epidemiological situation and notification of outbreaks.

According to EU legislation on ASF (Council Directive 2002/60/EC) [21], as soon as ASF is confirmed in wild boar in any MS, the competent authority shall immediately evaluate the epidemiological situation and define an infected area in order to apply the relevant control measures. Furthermore, the working document SANTE/7113/2015—Rev 12 [22] already envisaged a strategic and harmonious approach based on the epidemiological situation of ASF in the EU MS. This strategy was adapted to the local circumstances of the country or region of application (regionalization). With these new directives, the public veterinary authority was directly involved in the management of wild boar, in particular applying a combination of zoning and fencing. Focal fencing combined with hunting, carcass removal, trapping and culling policies has been useful in reducing or eliminating wild boar populations, thus achieving the eradication of ASF, as happened in Belgium and the Czech Republic in 2018 [22].

The AHL not only strengthens the roles of the official veterinary services to fight the disease, but also provides for (and formalizes) an explicit and “official” holistic approach for the management of animals (farms at risk and wild boar populations) and their environment. Veterinary services have to know and manage this issue in a new way, asking for data/information from an “ad hoc” appointed team of experts, along with the local authorities, and modulating the measures to be adopted according to the logistic characteristics of the infected area (sometimes requiring building artificial barriers, as in the case of Germany, Belgium, the Czech Republic and Italy). Official veterinarians must have up-to-date data on wildlife populations to monitor the efficiency of depopulation campaigns. This means that veterinary epidemiologists require a constant exchange of data with the categories of professionals and citizens dealing with wildlife (naturalists, rangers, hunters, etc.).

## 3. Discussion

According to the new regulation, official veterinary services have to plan and manage measures and restrictions applied to animal farms or the movements of swine products, using “risk-based approach” measures and restrictions applied to animal farms or movements of derived swine products. Moreover, for the first time they also must organize, manage, coordinate and monitor measures to be applied in the wild boar habitat. Among these measures, the AHL stresses the importance of collecting data on the size and ecology of wild animal populations (a task previously assigned to conservation agencies and/or wildlife management experts) in order to organize sustainable and effective depopulation campaigns of susceptible wildlife and improve passive surveillance. In other words, some activities which were previously the responsibilities of other regional authorities now fall within the responsibilities of the official veterinary services. This does not mean that official veterinarians must become experts in wildlife ecology or advanced counting techniques, but they must change their way of managing surveillance and control of animal diseases through active interaction with all stakeholders and/or experts who are functional to their objective. It is a matter of a new holistic approach which takes into account new alternative tools in order to prepare sustainable and effective actions (the so-called EU-recommended “*Preparedness*”) through an exchange of competences within regional/local technical boards, or simply through sharing databases of all essential information (geographical, zoological, ecological, social, etc.), which could represent a feedback of knowledge to build/plan surveillance and control activities.

CIR (EU) 2023/594 “Principles and criteria for geographically definition” describes the ASF infection risk and identifies a restricted zone surrounding the ASF-confirmed cases in order to prevent the spread of infection. With this approach, the legislator may act rapidly against infection at the outset of ASF. In the ASF-free regions’ neighboring infected areas with a strong potential risk of spreading the disease through wild boars, geographical areas called white zones (WZ) will be established, where all measures will be applied to reduce the wild boar population and, at the same time, enhance passive surveillance. Through these measures, the WZ is expected to act as a buffer to reduce the chances for the virus to infect susceptible animals and extend the contagion. The infected area (restriction zone II) is expected and requested to be left depopulated by the high mortality rate of ASFV (when spreading in a free area), which helps “auto limiting” of infection in the area [21].

Restriction zones are constantly amended and updated according to the evolution of the EU case notification, and subjected to particular and specific movement restrictions for animals, products and materials of animal origin. Correct configuration and application of restriction zones are mainly based on two factors: the geographical characteristics of the region and, in the case of wild boar involvement, the ecology of the species, considering the territorial impact related to their host range.

Derogation to allow animals and products to move throughout infected countries or across EU MS can be granted, according to the previously established risk evaluation approach and under the responsibility of the local competent authorities. The new regulations in the AHL are related to specific measures to be applied to wild habitats. Some of these regulations are similar to those applied in the pig farming system (such as to stop or deny any animal or product movement), while some others (such as establishing/modulating the geographical extension for public veterinary authority, communication with the potential observers or hunters of wild boars in the area and involving official vets in passive surveillance and monitoring of the success of the ASF eradication plan control) are new to official veterinarians, who are called to apply and modulate them in terms of intensity, considering all geographical, productive, cultural and social characteristics of the country involved. These characteristics denote that regulatory measures extend beyond activities of veterinarians and hunters to consider/involve the impact of stakeholder activities.

The application of the newly issued regulations in areas without borders requires the support and consultancies of “*non-veterinarian*” local actors such as police and other law enforcement agencies, forestry people, hunters, biologists, nature guides, etc., in order to accomplish the following tasks:

✓ Training of hunters to make them aware of the ASF-related risks and standard practices in case of finding suspected carcasses;
✓ Enhancement of passive surveillance (key measure) by all local actors;
✓ Implementation of depopulation actions to reduce wild boar density (mostly involving hunters and governmental designated operators);
✓ Setting up fences bordering the infected areas, which must be properly designed in robustness and size to limit the movements of wild boar;
✓ Control of the prohibition of any activity related to mushroom collection and/or fruit from the undergrowth;
✓ Regulation of human activities and movements inside the restriction zones.

Therefore, in the case of ASF, surveillance and control activities on susceptible wildlife need cooperation and the establishment of a network that connects public veterinary services to all other stakeholders and/or sectors working or living in wild areas. The additional measures to modulate the disease dynamics listed in the framework of the above-mentioned strategy (particularly the depopulation of wild boars) should be established in agreement with EU environmental, hunting and veterinary legislation (European and national), including ecosystem protection requirements and animal welfare. The intensity and size of measures may differ from one area to another, and control measures must be designed “ad hoc”, based on whether ASF has been identified or not in a particular region. The progress of the epizootic status of the disease and its different phases (incursion, invasion, epidemic and endemic) must be monitored using a constant flow of information obtained via passive surveillance and laboratory testing of carcasses and suspected animals originating from depopulation campaigns. Depopulating by hunting and exclusion of recolonization must be applied from outside the restriction zone (within which a naturally high mortality rate is expected) and must be performed centripetally from the external border of the buffer zones towards the infected zone. Both the fast and effective depopulation (top to bottom; in other words, starting from the periphery and continuing to the center) and the fencing (left to right, to push animals to move in one direction for efficient control) should support the efficiency of the measures [23]. Nevertheless, depopulated spaces may attract wild boar from surrounding populations in other areas, restocking the “at risk” zone and in this way maintaining the virus circulation indefinitely. For this reason, in order to get the outbreaks under control, veterinary authorities are authorized by EU regulations to create artificial barriers (often in combination with the existing ones) to surround the area, physically stopping the virus progression. In this way, the emergency zone can be fenced off to avoid any spillage of infected animals to the rest of the region [24].

The construction and maintenance of fences has resulted in successful eradication of ASF in some countries characterized by a single introduction of ASFV, such as Belgium (300 km long) [25]. On the contrary, in countries experiencing continued infectious pressure from neighboring countries, such as Germany, fences have likely slowed the spread of ASF, but could not achieve disease eradication.

In Italy, at the moment, we are observing a progressive, constant increase in new ASF outbreaks in the affected regions, suggesting that this kind of barrier may be effective in flat areas, as in Belgium, but will be less or poorly effective in orographically complex hilly or mountainous areas, which prevail in Italy, due to obvious difficulties in properly setting and maintaining the fences [26].

Maintenance is a critical issue to guarantee the long-term effectiveness of fences. Therefore, fences must be periodically inspected and repaired. Unfortunately, extensive fences, for example, at river crossings, are difficult to maintain and many vulnerable points appear [26]. Overall, evidence has shown that the strategy to fight against category A disease with a sylvatic reservoir is much more demanding when compared to the implementation of well-known good practices and biosafety rules in the farm environment.

To counteract ASF in boundless wild areas, it is necessary to activate an effective risk-communication campaign addressed not only to those actors who have responsibility in livestock management and animal-origin food production, but also to a range of other potential actors/stakeholders. This means that we need to inform all people involved in the management and attendance of natural parks and wild areas, to edit and produce digital or written documents (posters and brochures) and to stimulate enhanced passive surveillance by a public who is not familiar with veterinary issues.

Although the ASF control measures discussed in this article appear similar to those applied for the control of the classical swine fever (CSF) epidemic in EU MS in recent decades, in that case, the oral vaccination of wild boar against CSF represented the turning point in the fight against the disease (e.g., Germany between 1994 and 2008) [27]. Wild boar vaccination would also be the optimal strategy for the prevention and control of the current ASF epidemic in the EU MS. However, to date, there is no available vaccine against ASF with high immunoprotective potential, as inactivated vaccines have poor immune protection and available cell lines are not sufficient for efficient in vitro replication of ASFV [28]. In this context, the actions provided by the AHL in wildlife ecosystems represent unique methods to resist the spread of ASF in wild boar, at least until an effective vaccine is developed.

In our opinion, the newly introduced strategy of the AHL concerning introduction of veterinary measures into the wild is much more demanding and more expensive than those applied in the pig farming system environment. In the case of ASF, EU veterinary authorities may benefit from these proper, potentially efficient, clearly designed and sustainable control measures to manage the epidemiological risk in the wild.

With regard to the porcine industry, despite the presence of ASF in wild boar, the AHL allows a region to maintain commercial activity of products and live animals, even in the case of relatively close outbreaks, assuming proper application of farm biosafety measures. This new approach implies the highest epidemiological knowledge and responsibility by all actors in the animal production chain. Accordingly, raising awareness of infection risks and the means of prevention is required.

Finally, another point of strength of the AHL is its flexibility: all collected data on an ASF epizootic in EU MS, including data coming from wild boar depopulation campaigns, will allow the European Commission to improve and update the legislation constantly, in order to continuously design appropriate tools for disease prevention and hopefully, eradication.

## 4. Conclusions

Besides simplifying and strengthening the strategy already introduced by Art. 16 of the Directive 2002/60 CE, AHL explicitly assigns official veterinarians the responsibility of disease management, even in the wild environment. Furthermore, the new regulation asks official veterinarians to monitor the risk related to the size of wild populations, establishing operative networks with all other experts of animal ecology, in order to facilitate this “out of farm” activity. Wild boar play a key role in the maintenance and diffusion of disease, and the management of this species represents the greatest challenge in the control of ASF in the EU MS. ASF eradication may not always be an immediate, realistic goal when wild boar are the reservoir and oral vaccines are not available. Nevertheless, implementation of the AHL may be seen as an opportunity to understand the limitations of current EU-endorsed strategies in view of their desirable improvement. In our view, despite the well-known complexity of wildlife disease management issues, the AHL represents a solid legislative basis on which to articulate legislative measures to counteract ASF diffusion and limit its huge socio-economic impacts in EU MS.

## Data Availability

Data are contained within the article.
